# Comparative Analyses of the Digestive Tract Microbiota of New Guinean Passerine Birds

**DOI:** 10.3389/fmicb.2018.01830

**Published:** 2018-08-10

**Authors:** Kasun H. Bodawatta, Katerina Sam, Knud A. Jønsson, Michael Poulsen

**Affiliations:** ^1^Section for Ecology and Evolution, Department of Biology, University of Copenhagen, Copenhagen, Denmark; ^2^Section for Biosystematics, Natural History Museum of Denmark, Copenhagen, Denmark; ^3^Biology Centre AS CR v. v. i., Faculty of Science, Institute of Entomology and University of South Bohemia, Ceske Budejovice, Czechia

**Keywords:** symbiosis, microbiome, MiSeq amplicon sequencing, insectivores, omnivores, nutrition

## Abstract

The digestive tract microbiota (DTM) plays a plethora of functions that enable hosts to exploit novel niches. However, our understanding of the DTM of birds, particularly passerines, and the turnover of microbial communities along the digestive tract are limited. To better understand how passerine DTMs are assembled, and how the composition changes along the digestive tract, we investigated the DTM of seven different compartments along the digestive tract of nine New Guinean passerine bird species using Illumina MiSeq sequencing of the V4 region of the 16S rRNA. Overall, passerine DTMs were dominated by the phyla Firmicutes and Proteobacteria. We found bird species-specific DTM assemblages and the DTM of different compartments from the same species tended to cluster together. We also found a notable relationship between gut community similarity and feeding guilds (insectivores vs. omnivores). The dominant bacterial genera tended to differ between insectivores and omnivores, with insectivores mainly having lactic acid bacteria that may contribute to the breakdown of carbohydrates. Omnivorous DTMs were more diverse than insectivores and dominated by the bacterial phyla Proteobacteria and Tenericutes. These bacteria may contribute to nitrogen metabolism, and the diverse omnivorous DTMs may allow for more flexibility with varying food availability as these species have wider feeding niches. In well-sampled omnivorous species, the dominant bacterial genera changed along the digestive tracts, which was less prominent for insectivores. In conclusion, the DTMs of New Guinean passerines seem to be species specific and, at least in part, be shaped by bird diet. The sampling of DTM along the digestive tract improved capturing of a more complete set of members, with implications for our understanding of the interactions between symbiont and gut compartment functions.

## Introduction

Animal bodies are sophisticated centers of symbiotic interactions (Ley et al., 2008; Huttenhower and The human microbiome project consortium, 2012; Macke et al., 2017) that have allowed for adaptations enabling hosts to exploit new niches (Russell et al., 2009; Godoy-Vitorino et al., 2012; Dietrich et al., 2014; Macke et al., 2017). Of the many microbial communities associated with hosts, the digestive tract microbiota (DTM) is likely the most diverse, fulfilling essential functions in nutrition absorption, breakdown of macromolecules, vitamin synthesis, detoxification, and defense (Kohl, 2012; Poulsen et al., 2014; Ceja-Navarro et al., 2015). The DTM is not only of importance in the digestive tract, but also impacts the growth, development, and behavior of hosts (Round and Mazmanian, 2009; Sommer and Bäckhed, 2013). Although extensive work on DTMs has been carried out on a number of hosts, a thorough understanding of the composition and role of the DTM is limited to relatively few taxa, including mammals (Ley et al., 2008; Huttenhower and The human microbiome project consortium, 2012; Kohl et al., 2018), insects (Macke et al., 2017), and some captive and livestock animals (Waite and Taylor, 2015; Hird, 2017).

Despite the diversity and global distribution of birds, studies of their microbial communities are limited (Lee, 2015; Waite and Taylor, 2015), especially in wild populations (Hird, 2017). The majority of avian DTM studies have been performed on commercial animals and charismatic species such as penguins (order: Sphenisciformes, family: Spheniscidae), hoatzin (order: Opisthocomiformes, family: Opisthocomidae), kakapo (order: Psittaciformes, family: Strigopidae), and vultures (Order: Accipitriformes, family: Cathartidae and Accipitridae) (Godoy-Vitorino et al., 2012; Kohl, 2012; Dewar et al., 2013; Roggenbuck et al., 2014; Vela et al., 2015; Waite and Taylor, 2015). Although Passeriformes comprises more than 50% of bird diversity (~6,000 species) (Dickinson and Christidis, 2014), their DTM composition has only been explored in fewer than 100 species (e.g., Hird et al., 2015; Kropáčková et al., 2017; García-Amado et al., 2018). Passerine birds occupy a multitude of habitats and feeding guilds, making them an ideal group for investigation of the evolution and roles of DTM associations. This is also likely to provide a better understanding of how the DTM influences passerine richness, distribution, and environmental adaptations.

The assemblage of DTM may vary according to the function of each region in the digestive tract (Kohl et al., 2018). For example, the acidic stomach environment is one of the major regions of digestion of macromolecules, and would thus be expected to house a community of acidophilus bacteria that play roles in digestion. In contrast, the lower regions of the small intestine and large intestine likely harbor specialized bacteria that contribute to nutrient absorption and processing (Stevens and Hume, 1995; Lu and Domingo, 2008; Svihus, 2014). However, most avian DTM studies have been based only on cloacal swabs or fecal samples (Waite and Taylor, 2015), and these alone are unlikely to fully represent the microbial communities across the entire digestive tract. This assumption is supported by a few studies that have compared different digestive tract compartments and found differences in microbial community compositions (Videvall et al., 2017; Zhang et al., 2017; Drovetski et al., 2018; García-Amado et al., 2018). These studies demonstrated that the DTMs of cloacal or fecal samples may qualitatively represent the microbiota of other sections of the digestive tract but not quantitatively (Videvall et al., 2017; Zhang et al., 2017). Lu and Domingo (2008) also showed that the microbial genes in feces are skewed toward genes related to nutrient absorption and processing rather than to digestion. This indicates that the fecal microbiota may be overrepresented by bacteria in the last regions of the digestive tract, where nutrient metabolism takes place (Stevens and Hume, 1995). More comparative studies on the DTM of digestive tract compartments of wild birds may improve our understanding of what determines the composition of microbial communities in natural conditions and identify the bacterial symbionts that play important roles along the digestive tract. In this study we investigate multiple digestive tract compartments of nine New Guinean passerine bird species belonging to two feeding guilds (insectivores and omnivores); *Colluricincla megarhyncha, Ifrita kowaldi, Rhipidura atra, Crateroscelis robusta, Sericornis nouhuysi* (insectivores), *Melipotes fumigatus, Melanocharis nigra, Melanocharis versteri, Toxorhamphus poliopterus* (omnivores), and assess the appropriateness of investigating single compartments of the digestive tract to capture the full DTM diversity.

## Materials and methods

### Samples

**Figure 1:**
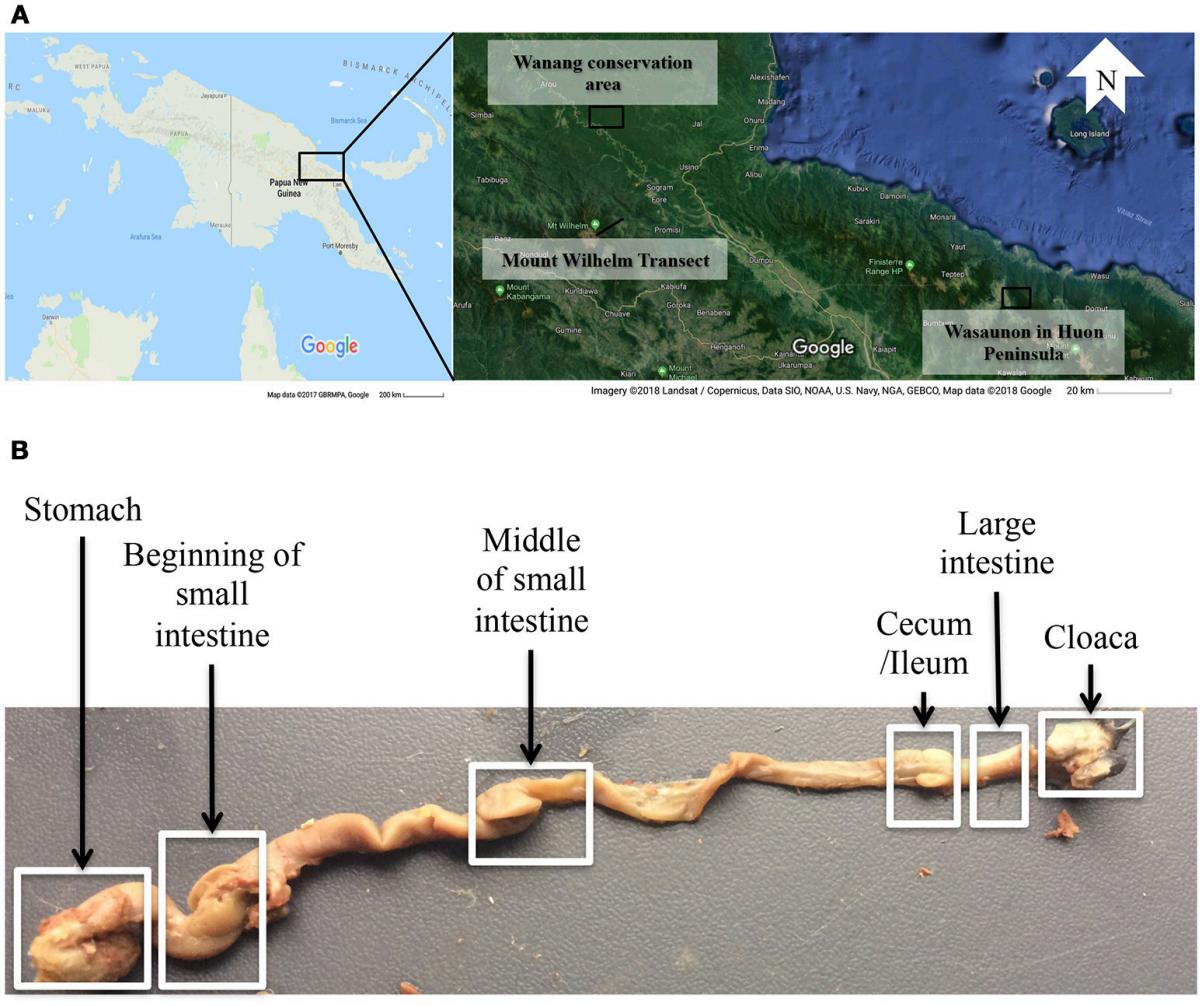
**(A)** A map of the sampling localities in Papua New Guinea: the Wanang conservation area, the Mount Wilhelm transect in Madang province, and Wasaunon on the Huon Peninsula in the Morobe province. **(B)** The digestive tract of *Melipotes fumigatus* indicating six compartments dissected from the alcohol specimen. The regurgitated samples were used to analyse the crop (not shown) microbiota.

A total of 33 alcohol bird specimens from the museum collections at the Natural History Museum of Denmark were used in this study. Twenty-nine specimens were collected in 2015 and 2016 in Madang Province in Papua New Guinea (PNG) at five elevations, ranging from 200 to 3,700 m a.s.l., along the Mount Wilhelm elevational transect. Three specimens were collected at Wasaunon (~2,950 m a.s.l.) in the Saruwaged Range on the Huon Peninsula in Morobe Province and one specimen was collected in the lowland rainforest of the Wanang Conservation Area (200 m a.s.l.) in Madang Province (Figure 1A, Table S1). Birds were captured using mist-nets, euthanized and immediately injected with 96% ethanol and preserved in 99% ethanol. An additional 51 regurgitated samples that had been collected between 2010 and 2012 from the same locations were included to analyse the crop microbiota. These samples were collected using the tartar emetic method to investigate the variation in diet of birds along the Mount Wilhelm elevational gradient, as described in detail by Sam et al. (2017). These samples were stored in 96% ethanol and select species were analyzed (Table 1 and Table S1).

**Table 1:** The nine Papua New Guinean passerine (Order: Passeriformes) bird species and number of original samples per sample type (R, regurgitated samples; A, dissected alcohol specimens), feeding guilds (Sam et al., 2017), and habitat and elevation (Pratt and Beehler, 2015).

**Family**	**Species**	**Species Code**	**R**	**A**	**Feeding Guild**	**Habitat**
Pachycephalidae	*Colluricincla megarhyncha*	Cm	3	2	Insectivorous	Lowland (0–1800 m)
Ifritidae	*Ifrita kowaldi*	Ik	3	1	Insectivorous (Toxic prey)	Highland (1450–2900 m)
Rhipiduridae	*Rhipidura atra*	Ra	6	4	Insectivorous	Highland (700–3200 m)
Acanthizidae	*Crateroscelis robusta*	Cr	6	4	Insectivorous (Some fruits)	Highland (1750–3600 m)
Pardalotidae	*Sericornis nouhuysi*	Sn	6	5	Insectivorous (Some fruits)	Highland (1200–3750 m)
Meliphagidae	*Melipotes fumigatus*	Mf	6	3	Omnivorous (Fruits + Insects)	Highland (1000–4200 m)
Melanocharitidae	*Melanocharis nigra*	Mn	6	3	Omnivorous (Fruits + Insects)	Lowland (0–1200)
Melanocharitidae	*Melanocharis versteri*	Mv	6	4	Omnivorous (Fruits + Insects)	Highland (1700–3680 m)
Melanocharitidae	*Toxorhamphus poliopterus*	Tp	6	6	Omnivorous (Nectar + Insects)	Highland (300–2450 m)

Digestive tracts were dissected from alcohol specimens of nine species (Table 1) to represent multiple passerine clades (Corvides, Passerides, and Meliphagides). One to six individuals of each species were included (Table 2) and the number of bird individuals per species was governed by the availability of alcohol specimens. Six segments (stomach, beginning of the small intestine, middle of the small intestine, ceca region and the ileum, large intestine, and cloacal region) (Figure 1B) of the digestive tract were used.

**Table 2:** The number of samples used for DNA extraction (S), samples that amplified during MiSeq sequencing (A) and samples that passed quality checks, filtering, and were deemed suitable based on visual inspection of the rarefaction curves (Figure S1) (Q).

	**Regurgitated samples (Crop)**			**Stomach**			**Beginning of small intestine**			**Middle of small intestine**			**Cecum/Ileum**			**Large intestine**			**Cloaca**		
**Species**	**S**	**A**	**Q**	**S**	**A**	**Q**	**S**	**A**	**Q**	**S**	**A**	**Q**	**S**	**A**	**Q**	**S**	**A**	**Q**	**S**	**A**	**Q**
*Ifrita kowaldi*	3	1	**1**	1	1	**1**	1	1	**1**	1	1	**1**	1	1	**1**	1	1	**1**	1	1	**1**
*Colluricincla megarhyncha*	3	2	**2**	2	2	**2**	2	2	**2**	2	2	**2**	2	2	**2**	2	2	**2**	2	2	**1**
*Rhipidura atra*	6	1	**1**	4	4	**4**	4	2	**1**	4	3	**3**	4	4	**4**	4	4	**4**	4	4	**4**
*Crateroscelis robusta*	6	1	**1**	4	4	**4**	4	2	**2**	4	4	**4**	4	4	**4**	4	4	**4**	4	4	**4**
*Sericornis nouhuysi*	6	4	**4**	5	3	**1**	5	4	**3**	5	5	**5**	5	5	**5**	5	5	**5**	5	4	**2**
*Melipotes fumigatus*	6	0	**0**	3	2	**0**	3	2	**2**	3	1	**1**	3	2	**2**	3	1	**1**	3	2	**2**
*Melanocharis nigra*	6	2	**2**	3	2	**1**	3	3	**3**	3	3	**2**	3	1	**1**	3	3	**2**	3	2	**1**
*Melanocharis versteri*	6	4	**4**	4	2	**2**	4	1	**1**	4	0	**0**	4	2	**2**	4	3	**2**	4	4	**3**
*Toxorhamphus poliopterus*	6	3	**4**	6	3	**3**	6	4	**4**	6	4	**4**	6	4	**4**	6	4	**3**	6	4	**1**

*Columns highlighted in bold font indicate the number of successfully-amplified samples*.

### DNA extraction, initial PCR, and MiSeq amplicon sequencing

DNA was extracted using DNeasy Blood and Tissue kits (Qiagen, Germany) following the protocol, except that only 75 μl AE buffer was used to elute the DNA. The amount of tissue varied from 15 to 30 mg depending on the compartment, and the extraction protocol was adjusted accordingly. Diagnostic PCR was conducted at the University of Copenhagen to confirm the presence of sufficient bacterial DNA. We used primers for the V4 region of the 16S rRNA gene (′V4.SA504 and ′V4.SB711) following Otani et al. (2016) and PCRs were conducted under conditions of 96°C for 4 min, 35 cycles of 94°C for 30 s, 56°C for 30 s, and 72°C for 30 s, and an extension step of 74°C for 4 min (cf., Otani et al., 2016). Amplifications were assessed on a 1.5% agarose gel.

Successfully amplified samples from the diagnostic PCR were sequenced by the Microbial System Molecular Biology lab at the University of Michigan using Illumina MiSeq. PCR reactions were 20 μl and the master mix contained AccuPrime PCR buffer 2, AccuPrime HiFi polymerase and 4 μM of the same primers (′V4.SA504 and ′V4.SB711). Reactions were performed under the PCR conditions of denaturing of 96°C for 1 min, 30 cycles of annealing (95°C for 20 s, 55°C 15 s, and 72°C for 5 min), and extension at 72°C for 10 min.

### Data analysis

MiSeq sequences were analyzed using Mothur 1.35.1 (Kozich et al., 2013). Chimeric sequences were filtered using the VSEARCH package and non-bacterial sequences (e.g., mitochondrial sequences) were removed. Operational Taxonomic Units (OTUs) (Cut-off 97%) of bacteria were aligned and identified using the SILVA bacterial reference library (Kozich et al., 2013). Sample coverage was evaluated by generating rarefaction curves with Mothur 1.35.1 (Figure S1). Samples with less than 2,500 reads and samples that did not display a flattening rarefaction curve based on visual inspection were excluded (Table 2). We also calculated the inverse Simpson's diversity index and the Bray-Curtis dissimilarity of bacterial communities in different compartments. We used vegan and Bidiversity R packages in R version 3.5.0 (R Core Team, 2013) to conduct statistical analysis. To visualize community similarities, we conducted a principal component analysis (PCA), and a scree plot analysis was conducted to evaluate the variation explained by the first ten principal components. We also conducted an adonis statistical test (a permutational multivariate analysis of variance using Bray-Curtis distance matrices) with 999 permutations to examine whether there were species-level and digestive tract compartment-level differences in DTMs. FigTree v1.4.3 (http://tree.bio.ed.ac.uk/software/figtree/) was used to generate trees.

## Results

After a chimera check and quality filtering, a total of 7,505,272 bacterial DNA sequences were retained from 149 intestinal compartment samples. The number of sequences per sample ranged from 2,904 to 261,625 (average 50,371), classified to 15,767 OTUs at the 97% sequence-similarity cut-off level (range 29–4,017 per sample; Table S2). Classified sequences belonged to 27 phyla, dominated by Firmicutes (49.2% of the OTUs), followed by Proteobacteria (25.6%) and Actinobacteria (12.7%). Only 5% of the sequences could not be classified at the phylum level (Table S3). The most abundant genera include an unclassified Carnobacteriaceae genus (Firmicutes) representing 31.4% of all sequences, followed by an unclassified Enterobacteriaceae genus (8%: Proteobacteria), *Lactobacillus* (4.9%: Firmicutes), an unclassified Gammaproteobacteria genus (4.7%: Proteobacteria), an unclassified Actinomycetales genus (2.6%: Actinobacteria), *Rothia* (2.5%: Actinobacteria), an unclassified Rhizobiales genus (1.9%: Proteobacteria), and an unclassified Mycoplasmataceae genus (1.6%: Tenericutes). We did not distinguish between intra- and extra-cellular bacteria in our approaches, but since we only found one well-represented genus of a (facultative) intracellular bacteria (*Bartonella*) in one individual of *R. atra*, we anticipate that the bacteria identified are most likely to primarily be extracellular.

**Figure 2:**
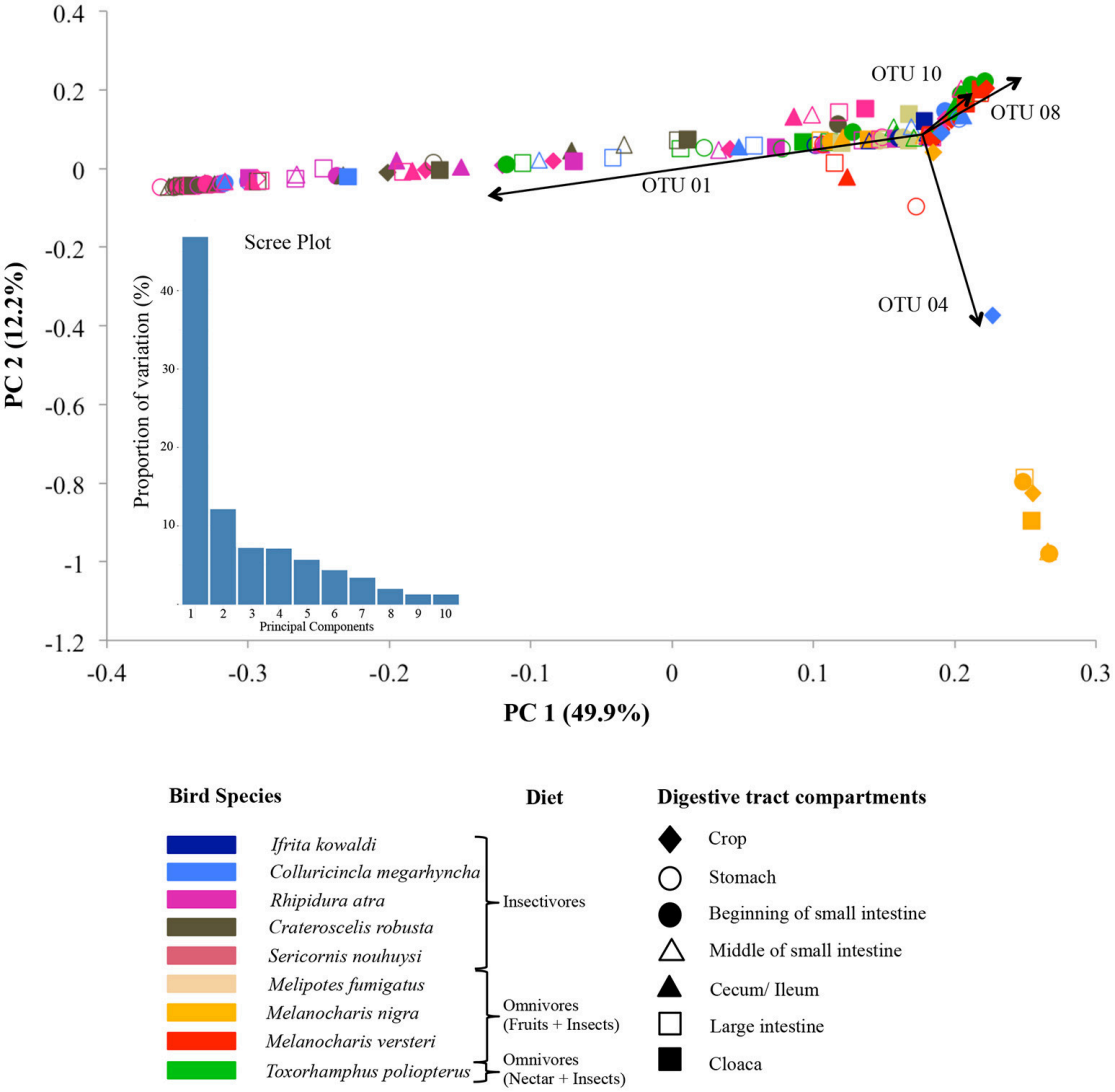
Principal component analysis (PCA) of DTM communities in different digestive tract compartments (shapes) in nine New Guinean passerine species (colors). The main dietary guilds of the bird species are indicated in front of their names in the figure key. The scree plot represents the variation in the data captured by the first 10 principal components. Arrows indicate the OTUs that mainly drive the microbial community separation: OTU 01 (unclassified Carnobacteriaceae genus, Firmicutes) and OTU 04 (*Lactobacillus*, Firmicutes), OTU 08 (unclassified Mycoplasmataceae genus, Tenericutes), and OTU 10 (*Helicobacter*, Proteobacteria).

**Figure 3:**
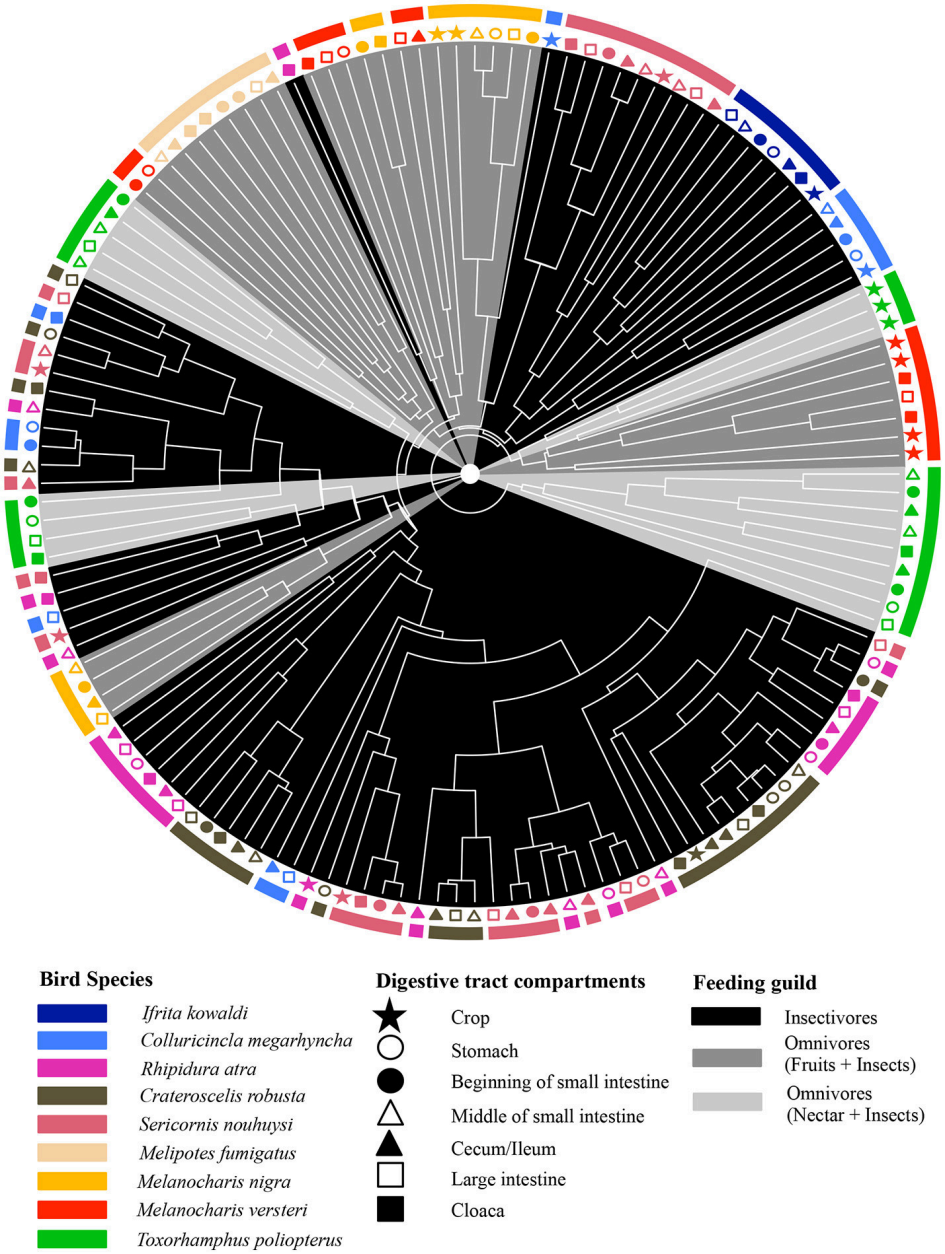
Dendrogram based on Bray-Curtis dissimilarity of DTMs between different compartments (different shapes) of the digestive tract of nine New Guinean passerine species (different colors). Gray scale shading of clades represents the feeding guilds of the bird species.

The adonis tests showed that DTMs were significantly different between bird species (*p* = 0.001, *R*^2^ = 0.24) but there were no significant difference between digestive tract compartments (*p* = 0.627, *R*^2^ = 0.04). Loading value analyses (Table S4) indicated that OTU 01 (Firmicutes, unclassified Carnobacteriaceae) contributed the most to the separation of *S. nouhuysi, C. robusta*, and *R. atra* communities (insectivores) in PCA space, while OTU 04 (Firmicutes, *Lactobacillus*) contributed to the separation of most *M. nigra* samples from the remaining communities. Furthermore, OTU 08 (Tenericutes, unclassified Mycoplasmataceae) and OTU 10 (Proteobacteria, *Helicobacter*) accounted for the separation of *M. versteri* and *T. poliopterus* (omnivores) from the others (Figure 2). Overall, Bray-Curtis distances revealed that bacterial communities in insectivorous species were more similar to each other than bacterial communities were to each other in omnivorous species (Figure 3). However, clustering was not in two perfectly distinct groups; rather, there were multiple clusters comprised of either insectivorous or omnivorous DTMs, and the omnivores were sub-clustered according to nectar feeders vs. fruit feeders (Figure 3). Different gut compartments of insectivorous species were more similar than those of omnivorous species, and compartments from the same individual were more similar to each other than to those of other individuals (Figures 2, 3).

**Figure 4:**
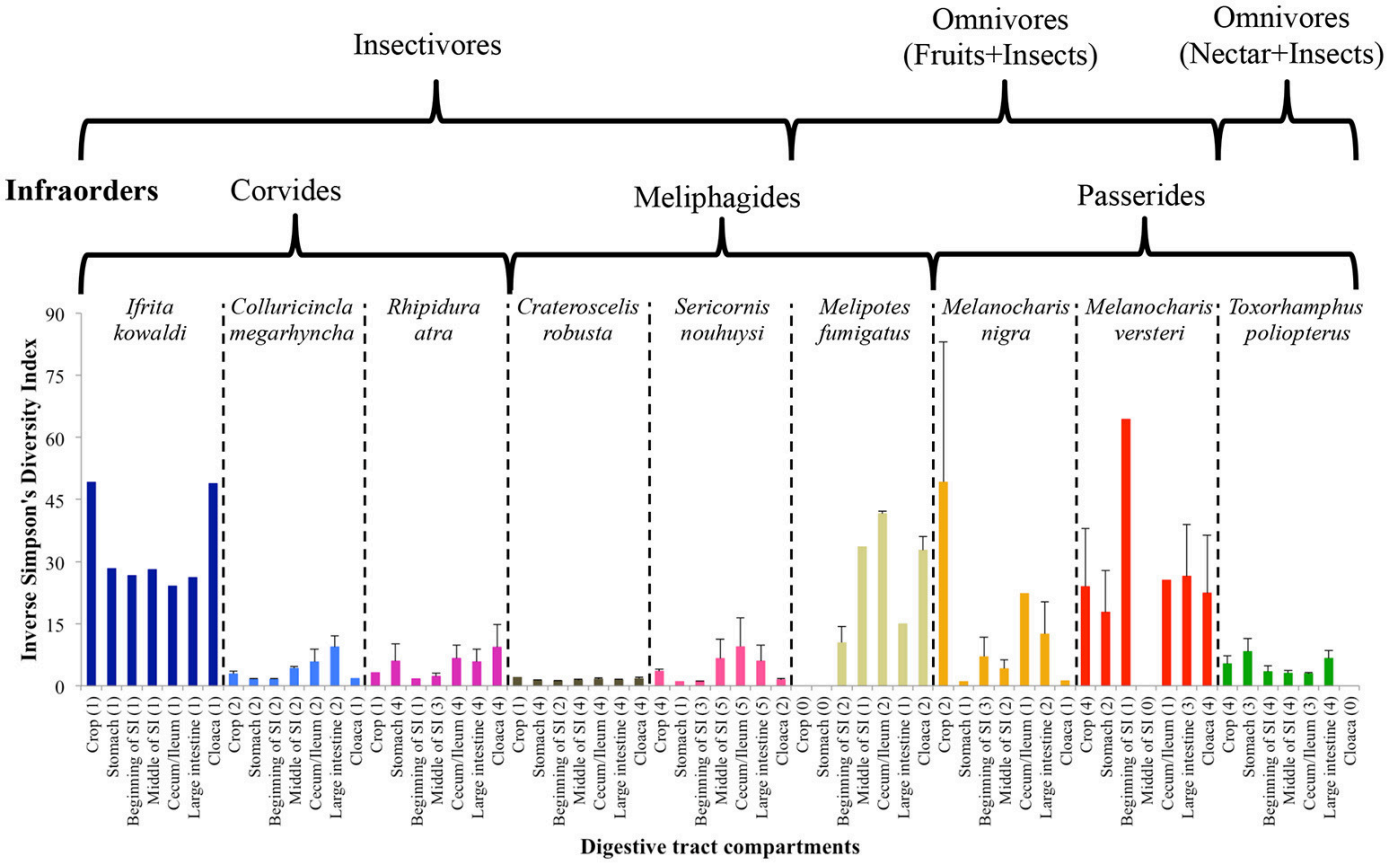
Average ± SE Inverse Simpson's diversity index of DTM in different digestive tract compartments. The infraorders of the species and the dietary guilds indicated above the figure. Numbers of replicates are given in parentheses.

The inverse Simpson's diversity index of the DTM between different compartments varied between bird species; however, variation, based on the standard errors, between individuals was low in well-sampled species (Figure 4). Generally, diversity increased as one moved along the digestive tract in most insectivorous species, which had lower diversity than omnivorous species, with the exception being *I. kowaldi*. This trend was less consistent for omnivorous species, but lower diversity was observed in the midgut of most species, except for *M. versteri*. Statistical tests for individual species was not possible due to the low sample sizes.

**Figure 5:**
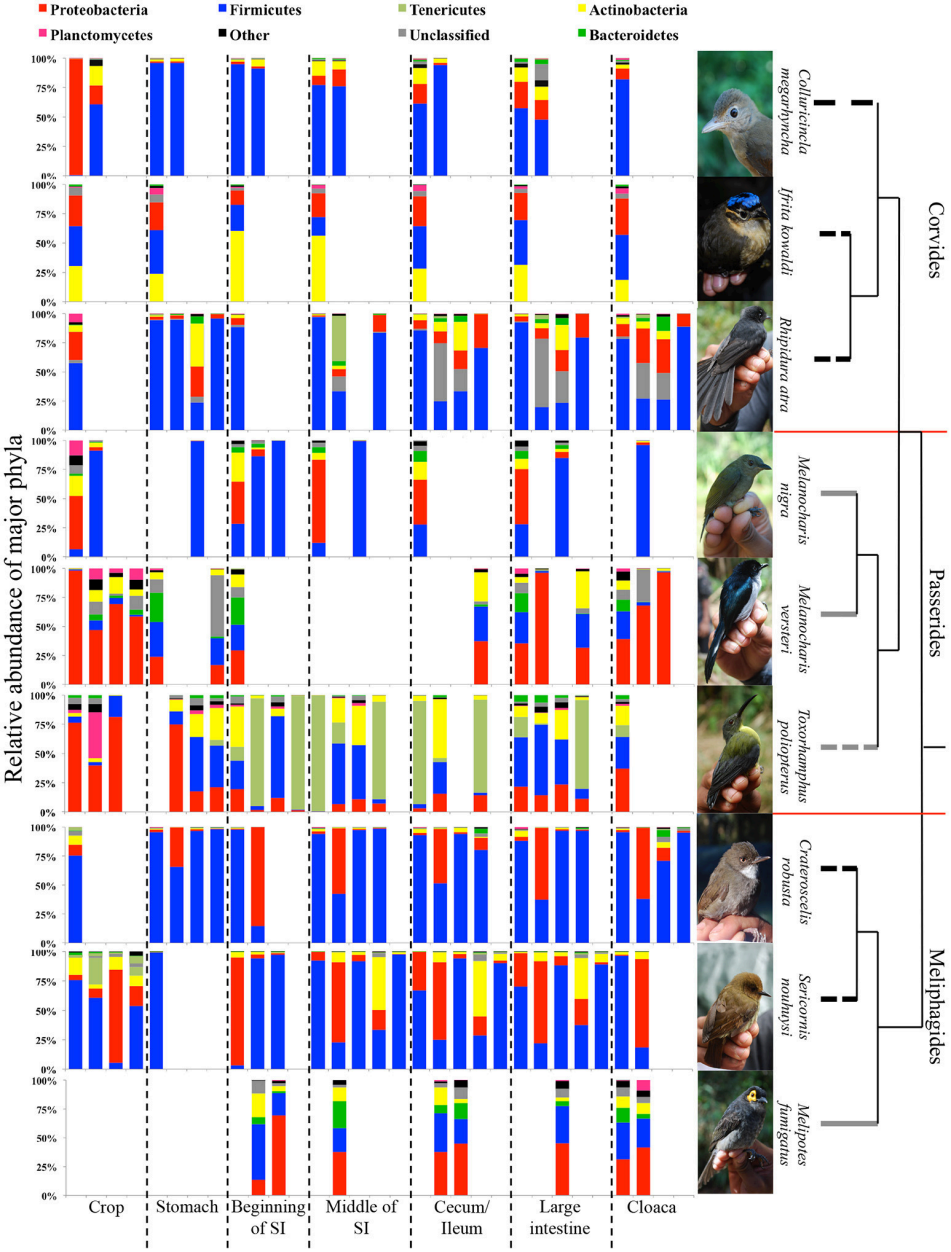
The proportion of major bacterial phyla present in the DTMs of different digestive tract compartments. The dendrogram represents the phylogenetic relationship between the bird species and the tips of the dendrogram represent the feeding guild of each species: black: insectivores, solid gray: omnivores (fruits and insects), and dashed gray: omnivores (nectar and insects). Red line indicates the separation of the bird infraorders. Photo credits: Andrew Hart Reeve.

Insectivores and omnivores displayed distinct differences in abundances of bacterial phyla. The DTM of insectivorous species were dominated by Firmicutes, except for *I. kowaldi*, which had a higher representation of Actinobacteria, whereas Proteobacteria dominated the DTM of omnivores. The nectar-feeding omnivore *T. poliopterus* had a higher number of OTUs belong to the Tenericutes. Furthermore, omnivores had a higher diversity of bacterial phyla compared to insectivores (Figure 5).

**Figure 6:**
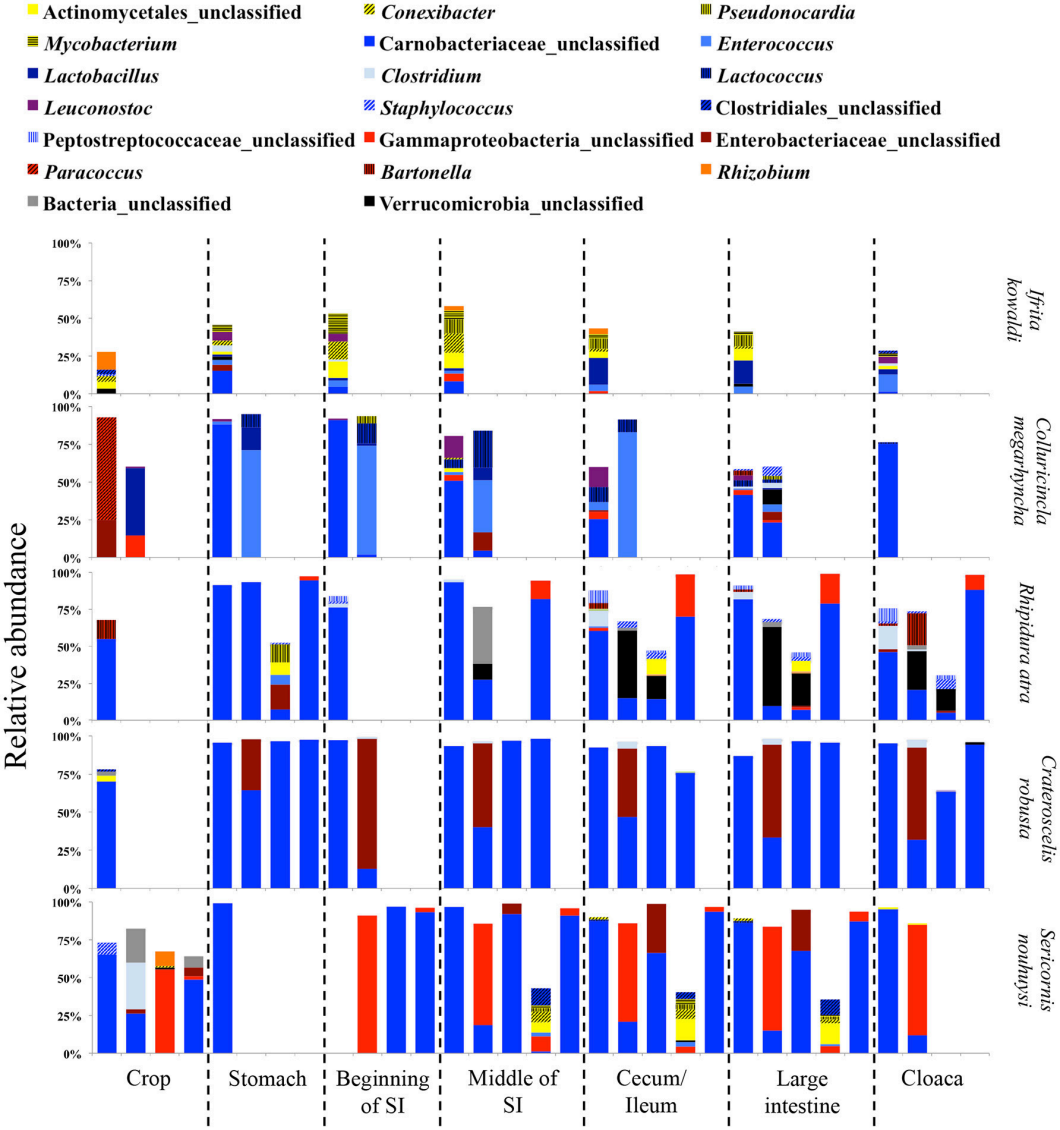
Relative abundance of the twenty most abundant bacterial genera in digestive tract compartments of insectivorous bird species. Bacterial genera belonging to the same phylum are represented by similar shades of colors.

**Figure 7:**
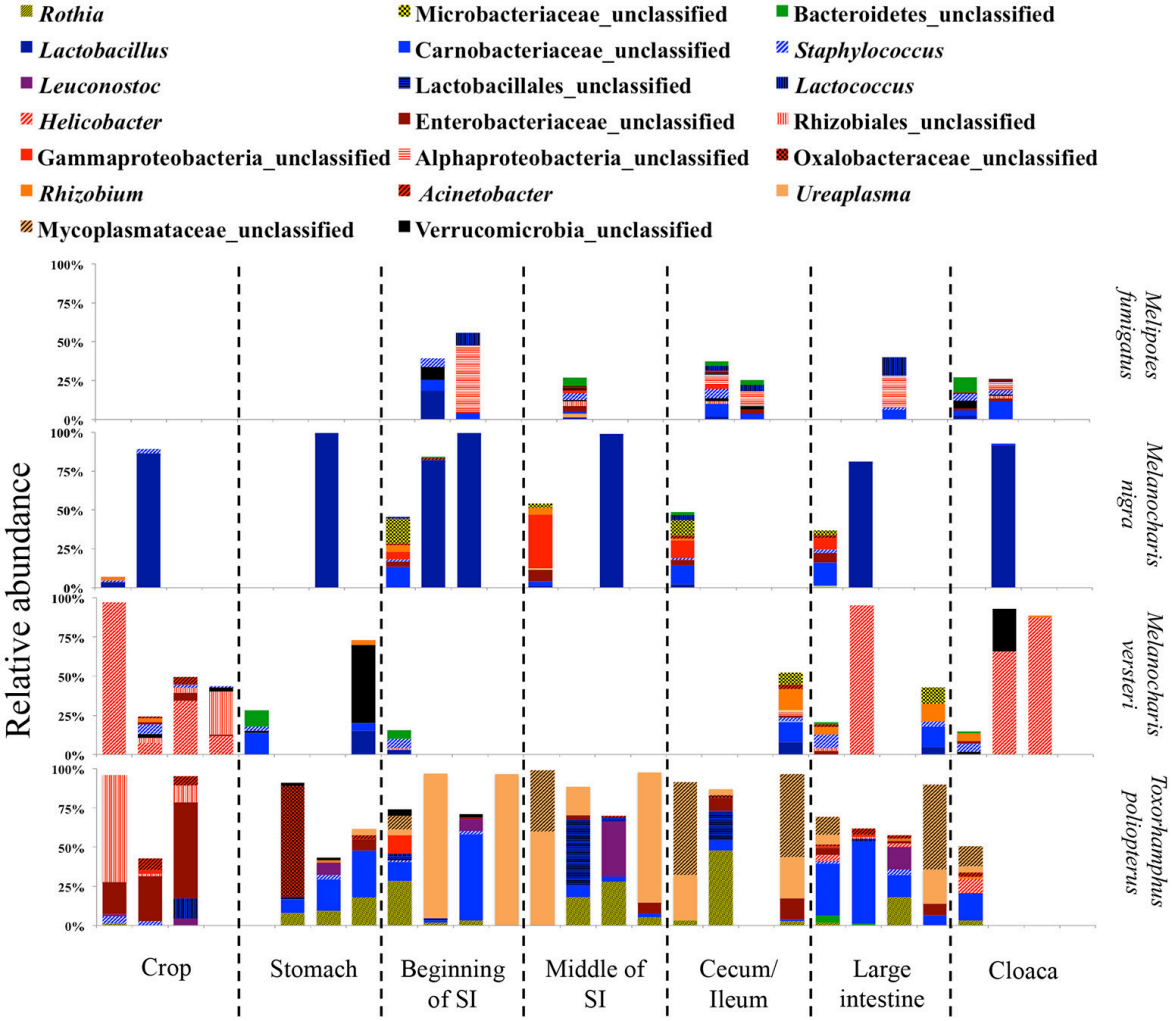
Relative abundance of the twenty most abundant bacterial genera in different digestive tract compartments of omnivorous bird species. Bacterial genera belonging to the same phylum are represented by similar shades of colors.

Due to the different abundances of bacterial phyla, we determined the 20 most abundant genera separately for each feeding guild, where only nine of the most abundant genera were shared (Table 3). Nine of the 20 most abundant genera in insectivores belonged to the Firmicutes (Figure 6). On average 75.2 ± 8.6% (SE) [83.4 ± 3.3% (SE) if we exclude *I. kowaldi*] of the OTUs belonged to the 20 most abundant genera, with the unclassified Carnobacteriaceae genus typically dominating. *I. kowaldi* appears to be a special case within the insectivores, with a more diverse and more evenly distributed microbiota. Consequently, only 42.6% of the sequences were covered by the 20 most abundant insectivorous genera. In omnivorous birds, eight out of the 20 most abundant genera were Proteobacteria and they represented 59 ± 9.7% (SE) of the OTUs, with the dominant bacterial genera varying between species. *M. nigra* was dominated by the genus *Lactobacillus, M. versteri* by *Helicobacter* and multiple other proteobacterial genera, the *T. poliopterus* DTM was mainly dominated by *Ureaplasma* and an unclassified genus in the Mycoplasmataceae, and the twenty most abundant genera were more evenly distributed in *M. fumigatus* (Figure 7).

**Table 3:** The 20 most abundant bacterial genera in insectivorous and omnivorous DTMs.

**Insectivores**			**Omnivores**		
**Phylum**	**Family**	**Genus**	**Phylum**	**Family**	**Genus**
Actinobacteria	Actinomycetales (U)	Actinomycetales (U)	Actinobacteria	Micrococcaceae	*Rothia*
Actinobacteria	Conexibacteraceae	*Conexibacter*	Actinobacteria	Microbacteriaceae	Microbacteriaceae (U)
Actinobacteria	Pseudonocardiaceae	*Pseudonocardia*	Bacteroidetes	Unclassified	*Unclassified*
Actinobacteria	Mycobacteriaceae	*Mycobacterium*	Firmicutes	Lactobacillaceae	*Lactobacillus*
Firmicutes	Carnobacteriaceae	Carnobacteriaceae (U)	Firmicutes	Carnobacteriaceae	Carnobacteriaceae (U)
Firmicutes	Enterococcaceae	*Enterococcus*	Firmicutes	Staphylococcaceae	*Staphylococcus*
Firmicutes	Lactobacillaceae	*Lactobacillus*	Firmicutes	Leuconostocaceae	*Leuconostoc*
Firmicutes	Clostridiaceae_1	*Clostridium*	Firmicutes	Lactobacillales (U)	Lactobacillales (U)
Firmicutes	Streptococcaceae	*Lactococcus*	Firmicutes	Streptococcaceae	*Lactococcus*
Firmicutes	Leuconostocaceae	*Leuconostoc*	Proteobacteria	Helicobacteraceae	*Helicobacter*
Firmicutes	Staphylococcaceae	*Staphylococcus*	Proteobacteria	Enterobacteriaceae	Enterobacteriaceae (U)
Firmicutes	Clostridiales (U)	Clostridiales (U)	Proteobacteria	Rhizobiales (U)	Rhizobiales (U)
Firmicutes	Peptostreptococcaceae	Peptostreptococcaceae (U)	Proteobacteria	Gammaproteobacteria (U)	Gammaproteobacteria (U)
Proteobacteria	Gammaproteobacteria	Gammaproteobacteria (U)	Proteobacteria	Alphaproteobacteria (U)	Alphaproteobacteria (U)
Proteobacteria	Enterobacteriaceae	Enterobacteriaceae (U)	Proteobacteria	Oxalobacteraceae	Oxalobacteraceae (U)
Proteobacteria	Rhodobacteraceae	*Paracoccus*	Proteobacteria	Rhizobiaceae	*Rhizobium*
Proteobacteria	Bartonellaceae	*Bartonella*	Proteobacteria	Moraxellaceae	*Acinetobacter*
Proteobacteria	Rhizobiaceae	*Rhizobium*	Tenericutes	Mycoplasmataceae	*Ureaplasma*
Unclassified	Unclassified	*Unclassified*	Tenericutes	Mycoplasmataceae	Mycoplasmataceae (U)
Verrucomicrobia	Verrucomicrobia (U)	Verrucomicrobia (U)	Verrucomicrobia	Verrucomicrobia (U)	Verrucomicrobia (U)

*Unclassified families and genera are represented with “U.” Gray highlighted genera presented in both insectivores and omnivores*.

In insectivorous species, the stomach and the beginning of the small intestine were typically dominated by 2–3 bacterial genera mainly belonging to Firmicutes (Figures 5, 6), while bacterial communities in the crop and the lower digestive tract were inhabited by more genera and phyla, such as Firmicutes, Proteobacteria, and Verrucomicrobia. In contrast, omnivores showed higher species-level and individual variation in the distribution of their 20 most common bacterial genera along the digestive tract (Figure 7). Moreover, the DTM of different compartments were typically dominated by multiple genera of Proteobacteria; although, the dominant genera tended to change along the digestive tract.

## Discussion

We investigated the DTM along the digestive tract of nine New Guinean passerine bird species and found that Firmicutes and Proteobacteria dominate the DTMs, which is consistent with previous studies of the passerine gut microbiota (Hird et al., 2014, 2015; Lewis et al., 2016; Kropáčková et al., 2017; García-Amado et al., 2018; Teyssier et al., 2018). DTM assemblages were species-specific and mainly shaped by diet, which agrees to some extent with recent studies on Neotropical (Hird et al., 2015; García-Amado et al., 2018) and European birds (Kropáčková et al., 2017). Even though we found species-specific DTMs, our microbial communities did not aggregate according to the phylogenetic placement of host birds; rather they clustered according to bird feeding guild. Previous studies on passerines have suggested that host phylogeny plays a main role in DTM assembly and ecological and life histories, such as feeding guilds, play secondary roles in shaping the DTM (Hird et al., 2015; Kropáčková et al., 2017; García-Amado et al., 2018). Our findings, however, are consistent with studies on mammals, for which host diet appears to be more important than host phylogeny (Ley et al., 2008; Muegge et al., 2011). Similar to previous DTM studies in passerines our study also demonstrates high individual variation in the DTM suggesting that the inclusion of more individuals is needed to assess the levels of intra-specific DTM variation.

The observed differences between the two feeding guilds (insectivores and omnivores) are most likely the result of differences in digestive needs (cf. Wang et al., 2016). Insectivorous diets are dominated by protein-rich arthropods, whereas omnivorous diets contain fruits, seeds, nectar, and insects (Sam et al., 2017). Lactic acid bacteria such as *Lactobacillus, Enterococcus*, and Carnobacteriaceae genera dominate the digestive tract compartments of insectivores. One of their main functions in the human digestive tract is carbohydrate metabolism (Hammes and Hertel, 2006; Pikuta, 2014), and a similar function is expected in birds. Many *Lactobacillus* and Carnobacteriaceae genera also produce antimicrobial substances that may play roles in the defense against antagonists, and they may play a role in detoxification through bile acid hydrolysis and removal of by-products of protein hydrolysis (Hammes and Hertel, 2006). *Enterococcus* bacteria can hydrolyse amino acids (LeBlanc, 2006), which has been shown to be important in carnivorous mammals to generate energy (Muegge et al., 2011). Thus, it is conceivable that insectivorous passerines with a protein-rich diet benefit in similar ways from lactic acid bacteria to improve energy uptake from protein-rich diets and by reducing harmful by-products of protein hydrolysis.

Omnivorous species, most of which had Proteobacteria-rich DTMs, were more variable in their DTMs between species than insectivorous birds. The majority of the OTUs driving these differences, e.g., Gammaproteobacteria (Enterobacteriaceae) and Alphaproteobacteria (Rhizobiaceae), include bacterial species that are capable of nitrogen fixation and amino acid synthesis (Panina et al., 2001; Zehr et al., 2003). The presence of these bacteria may thus contribute to the nitrogen budget of omnivorous birds, which feed on plant material that is generally low in nitrogen. Accordingly, Muegge et al. (2011) showed higher levels of amino acid synthesizing enzymes coded for by members of the microbiota in herbivorous mammals. A similar specialized DTM member function may apply for the nectar-feeding omnivore *T. poliopterus*. Nectar contains little nitrogen, so Mycoplasmataceae in the small and large intestines of *T. poliopterus* may help conserve nitrogen, as bacteria in this family are known to be able to hydrolyse urea (Glass et al., 2000). This is consistent with work by Preest et al. (2003), who suggested that uric acid may be broken down to re-cycle nitrogen in the lower intestine of nectar feeders, such as hummingbirds.

Some DTMs of the omnivorous *M. nigra* were more similar to those of insectivores, which is consistent with findings by Sam et al. (2017) that insects are a major component of the diet in some individuals of this species. Variable diets between individuals thus likely induce variation in DTMs. This is supported by the finding that the DTMs of some individuals of *M. nigra* are similar to insectivores, while others resemble omnivorous species. Comparable variability in DTM composition was recently documented in frugivorous Neotropical passerines (García-Amado et al., 2018), and a potentially more flexible and diverse DTM may enable omnivorous species to adjust their diet according to the availability of food resources. It appears a promising avenue of further research to determine how particular diets may alter the DTM composition for a given individual.

The DTM of *I. kowaldi*, which contains batrachotoxins in its tissue (possibly stemming from a diet of beetles, which also contain this toxin) (Dumbacher et al., 2000, 2008), was markedly different from all other insectivores in this study, with a higher proportion of Actinobacteria, including *Conexibacter* and *Pseudonocardia*. Bacteria from the genus *Pseudonocardia* inhabit toxic environments and typically have capacities to break down toxins (Veinberg et al., 2006), and members of the genus associate with fungus-farming ants for antimicrobial production (Cafaro et al., 2011). The genome of *Conexibacter* has a high proportion (11%) of genes related to amino acid metabolism (Pukall et al., 2010), suggesting a potential importance of *Conexibacter* for nitrogen recycling. Alternatively, the distinct composition of the DTM of *I. kowaldi* might be a result of its isolated phylogenetic position in the passerine phylogeny, within which it is the sole member of the family (Ifritidae) (Dickinson and Christidis, 2014). We were only able to include a single individual and were therefore unable to assess individual variation within this species. Future work comparing more individuals is clearly needed to corroborate our findings and to determine the reasons for the potentially relatively unique DTM composition of *I. kowaldi*.

We did not find common avian pathogenic bacterial genera, such as *Salmonella*, in our study. Some species belonging to common genera identified, such as *Lactobacillus, Enterococcus*, and *Staphylococcus*, can be pathogenic in birds (Benskin et al., 2009). However, these genera also contain a multitude of beneficial bacterial symbionts (Hammes and Hertel, 2006; LeBlanc, 2006).

Thus far, the majority of studies of avian DTM have focused on single compartments of the digestive tract to assess the composition of the entire digestive tract (Lee, 2015; Waite and Taylor, 2015) with evidence both for (Sekelja et al., 2012) and against (Lu and Domingo, 2008; Stanley et al., 2015) such an approach. We found notable species-specific differences in major bacterial groups along the digestive tract, with changes apparently depending on whether the birds were insectivores or omnivores. The crop region contained a higher DTM diversity and a different profile of bacteria compared to the mid gut regions. Some of this diversity might be accounted for by bacteria that entered with the food. We did not pursue this matter further but speculate that the reduced diversity and the changed composition of DTM in the stomach regions may be caused by the acidic stomach, which may act as a barrier to exogenous bacteria. Compared to the mid gut regions, the DTM in the large intestine and cloacal regions were more diverse and reflected the majority of bacterial OTUs from other regions of the digestive tract. The changes in DTM diversity along the passerine digestive tract and higher diversity in the hind regions, is consistent with a recent study on Deer mice (Kohl et al., 2018). Focusing on hind regions of the digestive tract thus likely provide sufficient qualitative insights into what microbes are present. However, they may not be sufficient to identify quantitative differences in major bacterial groups along the digestive tract, which align with previous comparative studies (Sekelja et al., 2012; Stanley et al., 2015; Videvall et al., 2017; Zhang et al., 2017). Moreover, the cloaca may also be affected by factors that mask the true endogenous DTM, such as the transfer of microbes during mating (White et al., 2010).

Our study was able to use museum alcohol specimens to investigate the DTM of multiple passerine species. This provides the first insights into New Guinean passerine DTMs and how DTMs change along the digestive tract of wild birds. We show that the DTM of insectivores is more consistent among species than omnivores and that the DTMs change along the digestive tract. The omnivorous DTMs were more variable but also show a change in major bacterial groups in different regions of the digestive tract. These differences in the DTM between feeding guilds appear to be driven by diet, where insectivores have a somewhat consistent diet based on insects, while omnivores have a more flexible diet including animal and plant material. Improved sampling of freshly dissected guts for an increased number of passerine species will enable a better understanding of the evolution of DTMs in this diverse bird order. It will also be relevant to explore different gut regions to improve our understanding of roles of these DTMs in different compartments based on the function of the digestive tract region.

## Data accessibility

MiSeq data is available from the NCBI SRA archive (SRP143623).

## Ethics statement

The fieldwork protocol, including ethical standards, was approved by the ethics committee at the Natural History Museum of Denmark. The fieldwork protocol follows Guidelines to the Use of Wild Birds in Research formulated by The Ornithological Council.

## Author contributions

KB dissected the digestive tracts and conducted the molecular work and sequence analyses. KJ collected bird specimens. KS collected the regurgitated samples. KB, KJ, and MP wrote the manuscript.

### Conflict of interest statement

The authors declare that the research was conducted in the absence of any commercial or financial relationships that could be construed as a potential conflict of interest.
