# Supplementary material for: Comparative Analyses of the Digestive Tract Microbiota of New Guinean Passerine Birds
Source: Front Microbiol. 2018 Aug 10;9:1830. doi: 10.3389/fmicb.2018.01830 (PMC6097311; doi:10.3389/fmicb.2018.01830)
Supplement: Supplementary file 1 [file Table_1.docx]

**Supplementary Material**

**Figure S1**: Rarefaction curves of all amplified samples. The number of reads on the x-axis is cut off at 2,500 to improve visibility. Any samples that had less than 2,500 reads or that did not have a flattening curve are labelled and these were removed from further analyses.

**Table S1:** Sampling locations of different bird specimens and regurgitated samples. Number of specimen belong to each species are shown in the parenthesis behind the species code. (Species codes: **Cm -** *Colluricincla megarhyncha,* **Ik** - *Ifrita kowaldi*, **Ra** - *Rhipidura atra*, **Cr** - *Crateroscelis robusta*, **Sn** - *Sericornis nouhuysi*, **Mf** - *Melipotes fumigatus*, **Mn** - *Melanocharis nigra*, **Mv** - *Melanocharis versteri*, **Tp** - *Toxorhamphus poliopterus*).

**Table S2:** Summary table of all samples sequenced in this study (separate excel file).

**Table S3:** OTU table of all the samples (full results from Mothur) with the taxonomic classifications of bacteria (separate excel file).

**Table S4.** Loading values for the PCA presented in Figure 1 generated using R 3.5.0 (separate excel file).

**Table S1.** Sampling locations of different bird specimen and regurgitated samples. Number of specimen belong to each species are shown in the parenthesis behind the species code (Species codes: **Cm -** *Colluricincla megarhyncha,* **Ik** - *Ifrita kowaldi*, **Ra** - *Rhipidura atra*, **Cr** - *Crateroscelis robusta*, **Sn** - *Sericornis nouhuysi*, **Mf** - *Melipotes fumigatus*, **Mn** - *Melanocharis nigra*, **Mv** - *Melanocharis versteri*, **Tp** - *Toxorhamphus poliopterus*).

| **Sampling location** | **Elevation (m)** | **Latitude** | **Longitude** | **Samples (A: Alcohol specimen, R: Regurgitated sample)** |
| --- | --- | --- | --- | --- |
| Kausi station (Mount Wilhelm transect) | ~200 | 05°44'33''S | 145°20'01''E | **A:** Mn (2), Cm (1)  **R:** Mn (1), Cm (2), |
| Numba station (Mount Wilhelm transect) | ~700 | 05°44'14''S | 145°16'12''E | **A:** N/A  **R:** Mn (2), Cm (1), |
| Memeku station (Mount Wilhelm transect) | ~1,200 | 05°43'18''S | 145°16'17''E | **A:** Mn (1), Tp (3), Cm (1)  **R:** Mn (3), Ra (1), Tp (1), |
| Deganumbu station (Mount Wilhelm transect) | ~1,700 | 05°45'45''S | 145°11'54''E | **A:** Ra (4), Cr (2), Sn (2), Mf (3), Mv (2), Tp (3)  **R:** Ra (4), Sn (2), Mv (1), Tp (4) |
| Bruno Sawmill station (Mount Wilhelm transect) | ~2,700 | 05°48'5''S | 145°09'02''E | **A:** Cr (1), Sn (2), Mv (2),  **R:** Cr (5), Sn (4), Mf (3), Mv (5), Tp (1), Ik (3), Ra (1), Mf (3) |
| Huon Peninsula (Wasaunon) | ~2,900 | 06°05'43''S | 146°54'57''E | **A:** Sn (1), Cr (1), Ik (1)  **R:** N/A |
| Wanang conservation area (Swire station) | ~200 | 05°13'39''S | 145°04'46''E | **A:** Cm (1)  **R:** N/A |
