# Supplementary figures and images for: Comparative Analyses of the Digestive Tract Microbiota of New Guinean Passerine Birds
Source: Front Microbiol. 2018 Aug 10;9:1830. doi: 10.3389/fmicb.2018.01830 (PMC6097311; doi:10.3389/fmicb.2018.01830)

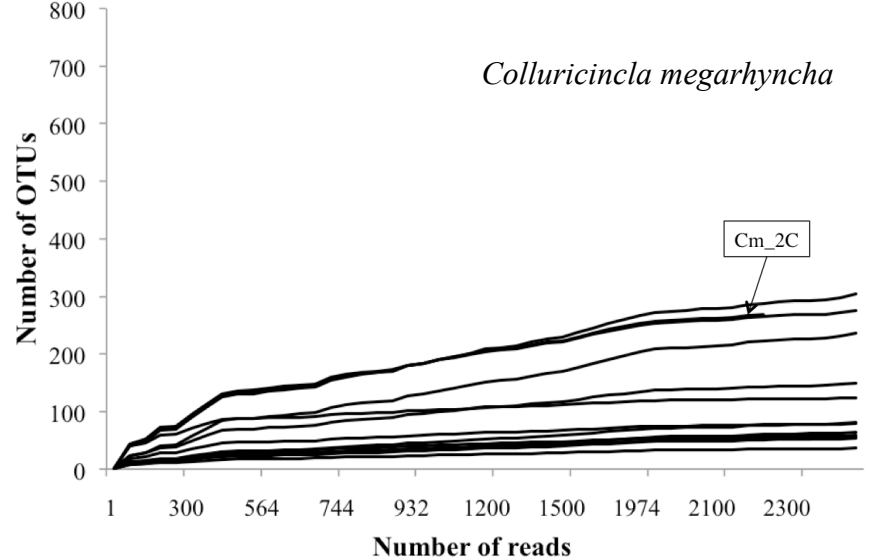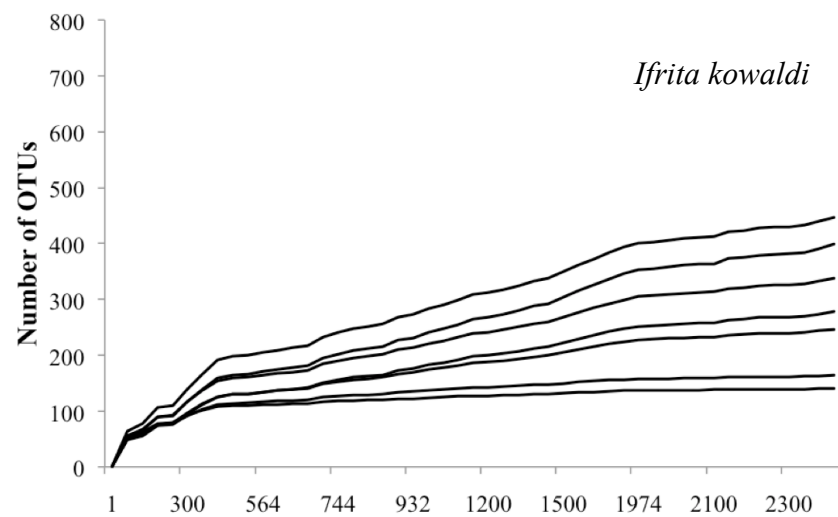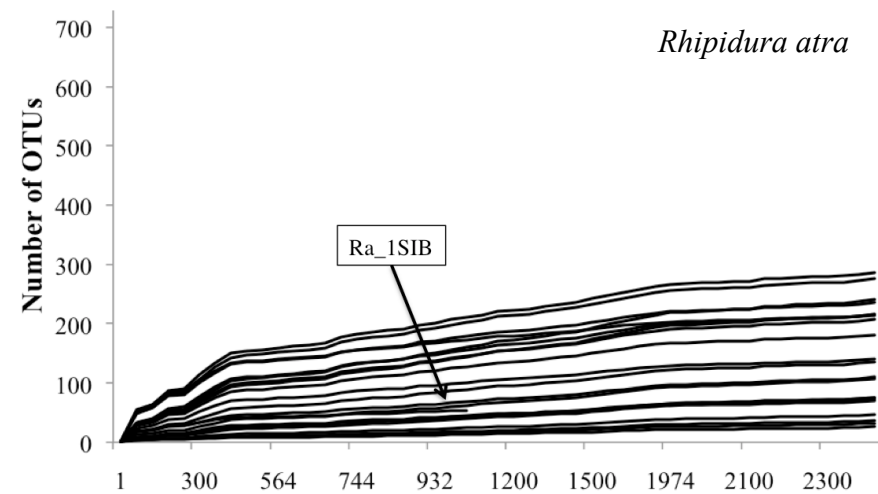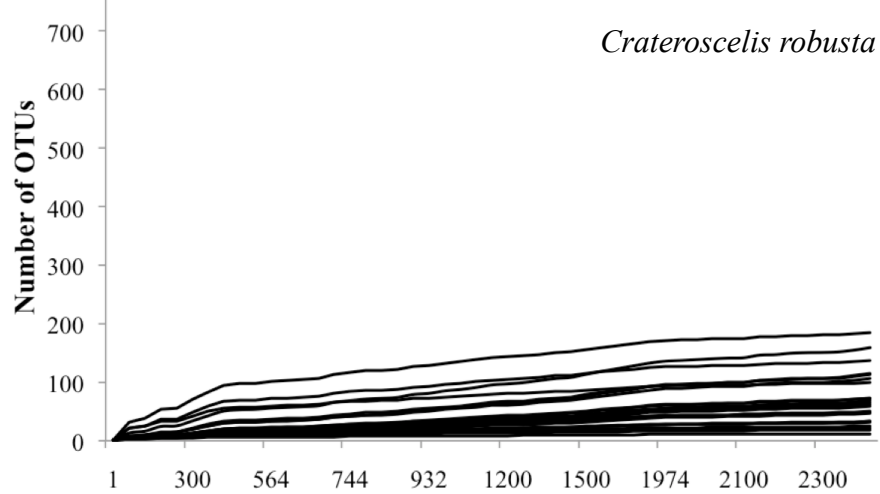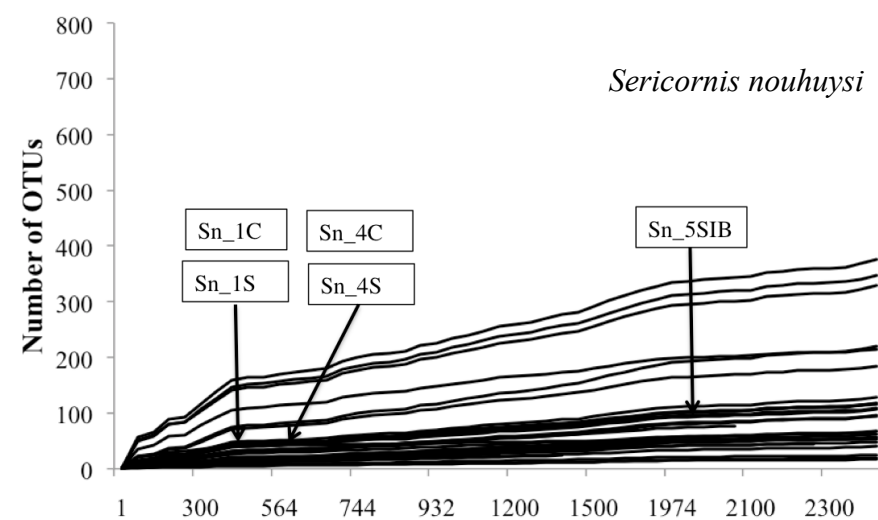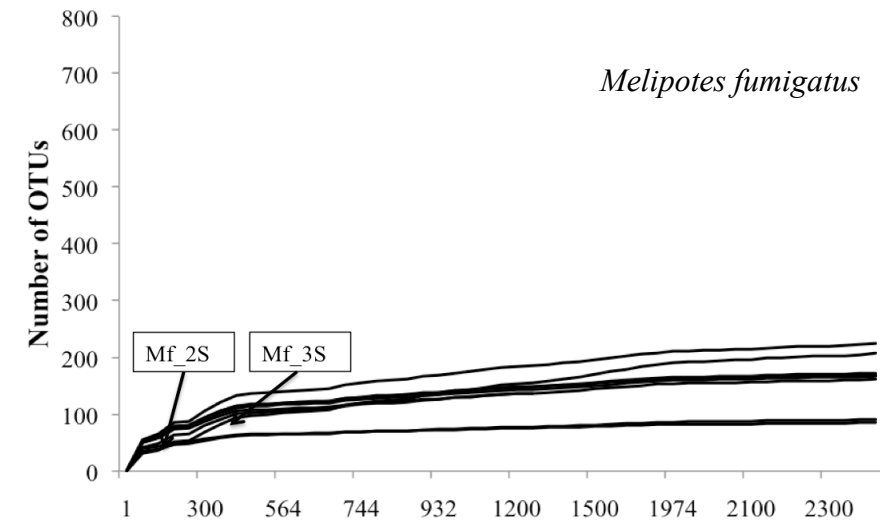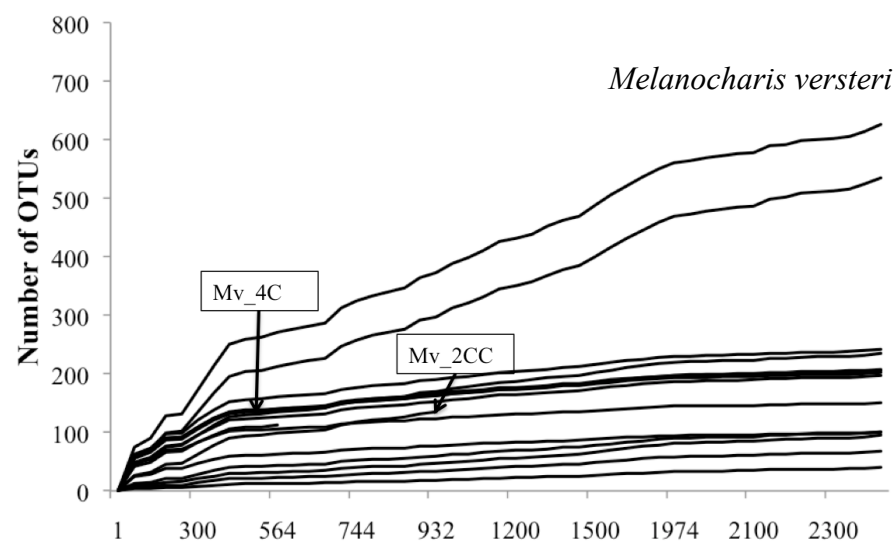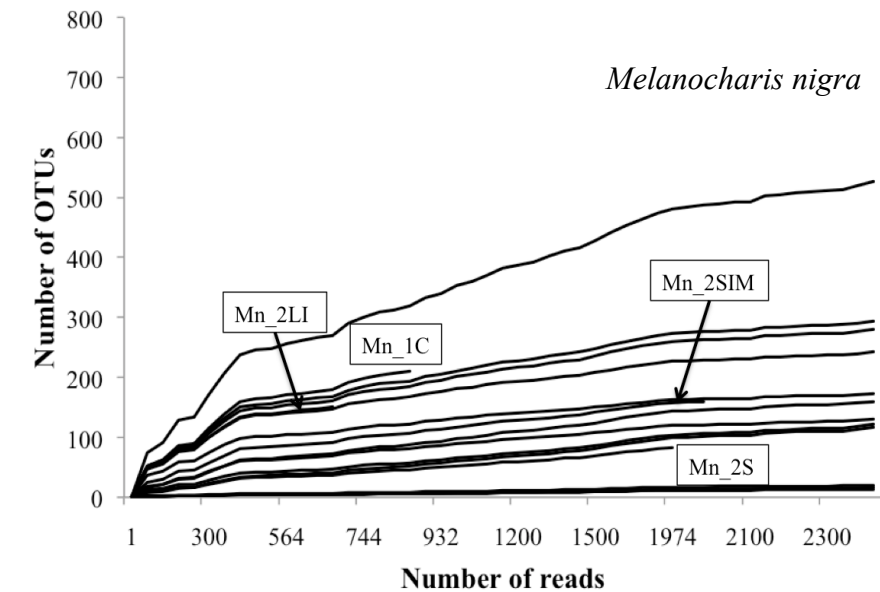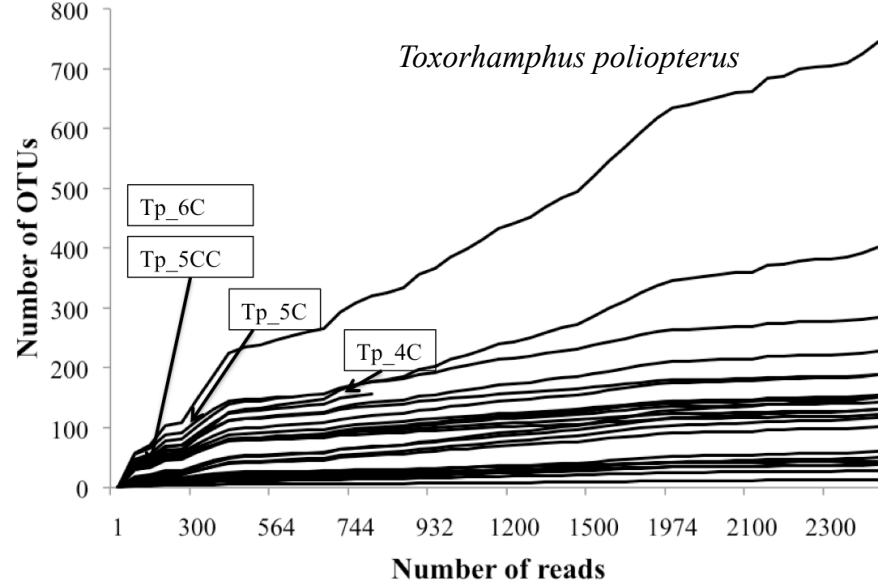

Supplement: Supplementary file 3 [file Data_Sheet_2.PDF]
